# Relation between Halls’ Professionalism Scale and nurses’ demographic characteristics

**DOI:** 10.1186/s13104-021-05660-2

**Published:** 2021-07-05

**Authors:** Tinda Rabie

**Affiliations:** NuMIQ Focus Area, School of Nursing Sciences, Faculty of Health Sciences, North-West University (Potchefstroom Campus), Noordbrug, Bult, P.O. Box 19389, Potchefstroom, 2522 South Africa

**Keywords:** Professionalism, Professional nurses, Demographic variables

## Abstract

**Objective:**

Nursing Professionalism was measured by Hall’s Professionalism Scale, consisting of 50 items. The scale was developed to measure the attitudes and ideologies held by professionals in various professional occupations by measuring five attitudinal constructs of professionalism, namely ‘sense of calling to the field’, ‘autonomy’, ‘using a professional organisation as a major referent’, ‘belief in self-regulation’, and ‘belief in public service’. This study focussed on determining the practically significant differences that exist between the means of the five constructs of Hall’s Professionalism Scale and certain demographic variables among nurses in South Africa. The 11-item demographic profile included the following variables: gender (1), age (2), age when becoming a professional nurse (3), undergraduate qualifications (4), marital status (5), number of children (6), employment sector (7), years’ of nursing experience (8), international experience (9), employment status (10) and satisfaction with nursing as a career (11).

**Results:**

Only (7/11) demographic profile variables had an association with one or more of the five Hall’s Professionalism Scale constructs The variables included the following items: age (2), age when becoming a professional nurse (3), number of children (6), years of nursing experience (8), international experience (9), employment status (10), and satisfaction with nursing as a career (11).

## Introduction

Nursing professionalism has a value-based foundation and is important to the success of healthcare services and standards and the delivery of quality patient care [1]. The concept of professionalism is defined by a code of conduct, professional relationships, competence, and communication abilities [1]. Other authors elicited the information that nursing professionalism is determined by cognitive, attitudinal and psychomotor characteristics, and important predecessor concepts of such characteristics include demographic, experiential, educational, situational and attitudinal aspects [2].

This study is part of a project with two objectives: (1) to determine the construct validity and internal consistency of Hall’s professionalism scale (HPS) tested on South African nurses [1]; (2) to determine the practically significant differences that exist between the means of the five constructs of HPS and certain demographic variables. The same questionnaire’s data set was used for both objectives, i.e. Section A—demographic profile and Section B—the 50 items of HPS. The HPS was developed by Professor Richard Hall, to determine the professional standing of an individual [3]. The HPS measures professionalism in five constructs, namely: using a professional organisation as a major referent, belief in public service, belief in self-regulation, sense of calling to the field, and autonomy—using a 5-point Likert scale with the options, very well (VW), well (W), neutral opinion (?), poorly (P) and very poorly (VP) [4, 5]. Originally, the HPS 50-item scale was tested on 328 participants, including nurses. The Cronbach’s alpha was 0.86, and Snizek’s shorter version, which included 25 of the original 50 items, yielded a Cronbach’s alpha of 0.79 [5].

Nursing is the largest group of personnel in the healthcare sector and plays an important role in the success of the healthcare system [6]. However, globally, nursing is also one of the most demanding professions, owing to long working hours, new technologies, budget constraints and an ever-changing healthcare environment [7]. Other well-known factors that contribute to the onerous demands of the profession include: staff shortages, increased care demands and the global burden of disease [8]. In addition, in developing countries such as South Africa, the healthcare sector is confronted with a global financial crisis and unemployment, leading to limited finance, poor resources and patient overcrowding, especially in the public healthcare sector [9]. These factors would inevitably affect professionalism demonstrated by nurses in the profession. It is also true that professionalism among nurses is affected by many other variables which are not as often researched, such as nurses’ demography [6, 10–16]. In the South African context, no previous study has been conducted which has specifically determined the practically significant differences between constructs of HPS and certain demographic variables.

## Main text

### Methods

This study used a preliminary quantitative, cross-sectional, correlational survey design. During 2017, data were collected using the online survey platform, Survey Monkey. An all-inclusive sampling of nurses (N = 2 158; n = 166) who were registered at the time or who had completed their undergraduate and/or postgraduate studies (2010–2017) at a specific university, was included. All participants received a computerised short message system (SMS) message with the following message: ‘Dear Mr/Mrs/Miss, you are invited to participate in a web-based 50-item survey based on the professionalism of nurses in nursing practice environments in South Africa. Should you decide to participate, kindly follow the http://-link [4]. Three SMSs were sent to each participant over days 1, 2 and 6. If the participant decided to participate in the study, he/she opened an http://-link that went to an introductory page and an informed-consent form. Those who decided to participate clicked on the agreement box that appeared on the screen and continued to the questionnaires.

The SPSS Version 25 computer program was used for data analysis, which consisted of three steps. Step 1, included descriptive statistics for the demographic profile and factor analysis of the HPS constructs to determine the Cronbach alpha. Step 2, tested associations of demographic variables with validated HPS constructs in the South African context, namely: ‘using professional organization as a major referent’, ‘belief in the public service’, ‘belief in self-regulation’, ‘sense of calling to the field’ and ‘autonomy’ [4]. t-tests, anovas, Cohen’s d values and Spearman’s rank order correlation were used. In step 3, Spearman’s rank order correlation was used to test the relationship between the HPS constructs. Because an all-inclusive availability sample random sampling was used instead of random sampling, p-values were irrelevant but were reported for completeness, and effect sizes were used to determine the importance of associations in practice. Cohen’s d value will be used with guidelines for interpretation of 0.2 as a small effect size, 0.5 as medium and 0.8 as large. Correlations of 0.1 were regarded as small, 0.3 as medium and 0.5 as large.

## Results

The results were presented in three steps. Step 1, included the demographic profile and descriptive statistics of the HPS constructs; step 2, described the association of demographic variables with HPS constructs; and step 3, described the correlation between the HPS constructs.

### Step 1: Demographic profile and desriptive statistics of HPS constructs (Table 1)

**Table 1:** Demographic profile and descriptive statistics of HPS constructs

Item numbers	Demographic variables	Percentages
Item 1	Gender	
	Male	11.45
	Female	88.55
Item 2	Age	
	Less than 30	24.70
	31–39 years	29.52
	40–49 years	31.33
	50 +	14.45
Item 3	Age becoming a professional nurse	
	< 25 years	70.48
	25–29 years	19.28
	30 + years	10.24
Item 4	Undergraduate qualifications	
	Baccalaureate degree	34.34
	Diploma	65.66
Item 5	Marital status	
	Single	49.40
	Married	50.60
Item 6	Number of children	
	1	24.17
	2	45.00
	3 +	30.83
Item 7	Employment sector	
	Public health care sector	59.04
	Private health care sector	40.96
Item 8	Years’ nursing experience	
	< 1	4.82
	2–3	6.63
	4–5	12.05
	6–10	15.06
	11–15	16.87
	16–24	17.47
	25–30	21.08
	31–35	3.61
	36 +	2.41
Item 9	International experience	
	Yes	7.83
	No	92.17
Item 10	Employment status	
	Full time	95.18
	Temporary	4.82
Item 11	Satisfaction with nursing as a career	
	Yes	79.52
	No	20.48

Constructs	Cronbach alpha	M	SD
Cronbach alpha, mean (M) and standard deviations (SD) of HPS constructs			
Autonomy	0.66	2.71	0.76
Sense of calling to the field	0.72	2.53	0.69
Belief in self-regulation	0.63	2.71	0.75
Using professional organization as a major referent	0.52	2.86	1.03
Belief in public service	0.52	1.97	0.63

Of the N = 166 nurses, the majority (147–88.55%) were females. Most participants (54.22%) were younger than 40 years of age. Most (70.48%) were registered nurses before 25 years of age. Most nurses (65.66%) had diplomas in nursing. Most of the nurses (50.60%) were married and just under half (49.40%) were single. Most (45%) had two children and less than a third (30.83%) had three or more children. The majority of nurses (59.04%) were employed in the public sector and most (55.42%) had between 11 and 30 years’ work experience. Most (92.17%) had no international experience, and the great majority (95.18%) was in full-time employment. Most of the nurses (79.52%) were satisfied with nursing as a career.

The construct, ‘belief in public service’, had the highest mean level (M = 1.97, SD = 0.63), followed by ‘sense of calling to the field’ (M = 2.53, SD = 0.69). ‘autonomy’ (M = 2.71, SD = 0.76) and ‘belief in self-regulation’ (M = 2.71, SD = 0.75) had the same mean; ‘using professional organization as a major referent’ (M = 2.86, SD = 1.03) had the lowest mean.

### Step 2: Association of demographic variables with HPS constructs

The association of demographic variables with HPS was done by using t-tests, anovas, Cohen’s d values and Spearman's rank order correlation. In this section, only statistically or practically significant associations were discussed.

#### Item 1: gender

Gender had no statistical/practical association with any of the five HPS constructs, (small Cohen’s d < 0.2, p ≥ 0.05).

#### Item 2: age

Although not statistically significant, persons 50 years of age and older (M = 2.38, SD = 0.66) agreed in practice that they had more ‘belief in self-regulation’ than those in the age groups 40–49 years (M = 2.71, SD = 0.78), 31–39 years (M = 2.77, SD = 0.66) and 30 years of age and less (M = 2.81, SD = 0.82). Cohen’s d value ranged between 0.43 and 0.59; (p > 0.05).

#### Item 3: age when becoming a professional  nurse

Persons who took up a position as a professional nurse at 30 years of age and older were more ‘autonomous’ (M = 2.44, SD = 0.70) than those who had become a professional nurse between 25 and 29 years of age (M = 2.61, SD = 0.84) or those younger than 25 years of age (M = 2.77, SD = 0.74), Cohen’s d value ranged between 0.21 and 0.45 (p > 0.05). Those who became professional  nurses at 30 years of age and older had a better ‘sense of calling to the field’ (M = 2.13, SD = 0.51) than those who had taken up nursing between 25 and 29 years of age (M = 2.55, SD = 0.74) and those younger than 25 years of age (M = 2.58, SD = 0.74). Cohen’s d value ranged between 0.66 and 0.57 (p > 0.05). Those who took up nursing at 30 years of age and older had a better ‘belief in public service’ (M = 1.71, SD = 0.50) than those who became nurses between 25 and 29 years of age (M = 2.07, SD = 0.73) or at younger than 25 years of age (M = 1.98, SD = 0.62). Cohen’s d value ranged between 0.43 and 0.49 (p > 0.05).

#### Item 4: undergraduate qualifications

Undergraduate qualifications had no statistical/practical association with any of the five constructs. Cohen’s d values ranged between 0.02 and 0.19 (p > 0.05).

#### Item 5: marital status

‘Marital status’ also indicated no statistical/practical association with any of the five constructs. Cohen’s d values ranged between 0.07 and 0.27 (p > 0.05).

#### Item 6: number of children

Nurses with two (M = 1.88, SD = 0.58), three, or more children had a higher ‘belief in public service’ (M = 1.85, SD = 0.54) than those with only one child (M = 2.37, SD = 0.64). Cohen’s d values ranged between 0.81 and 0.77 (p > 0.05).

#### Item 7: employment sector

Neither the public nor the private healthcare sector had a statistical/practical association with any of the five constructs. Cohen’s d value ranged between 0.06 and 0.24 (p > 0.05).

#### Item 8: years’ nursing experience

Spearman’s rank order correlation indicated that the years of professional nursing experience was directly proportional to the ‘belief in self-regulation’ (r = -0.157*).

#### Item 9: international experience

The constructs showed no statistical significance (p > 0.05). Nurses with no international experience had the highest ‘autonomy’ (M = 2.68, SD = 0.75; Cohen’s d value 0.48; p > 0.05), while nurses with international experience professed ‘using professional organization as a major referent’ (M = 2.23, SD = 0.86) more than did those without international experience. Cohen’s d value was 0.66 (p > 0.05).

#### Item 10: employment status

The constructs had no statistical significance (p > 0.05). Temporary nurses indicated that they had more ‘autonomy’ (M = 2.33, SD = 0.64, Cohen’s d value 0.52) and a higher ‘belief in self-regulation’ (M = 2.29, SD = 0.92, Cohen’s d value 0.49) than did full-time employees.

#### Item 11: satisfaction with nursing as a career

Nurses who were satisfied with a nursing career had a higher ‘sense of calling to the field’ (M = 2.44, SD = 0.66, Cohen’s d value 0.58; p > 0.05) and ‘belief in public service’ (M = 1.86, SD = 0.54) than those who were dissatisfied (M = 2.41, SD = 0.77), which was statistically significant (Cohen’s d value 0.72; p = 0.01).

### Step 3: Correlation between HPS constructs (Table 2)

**Table 2:** Correlation between HPS constructs

	Autonomy	Sense of calling to the field	Belief in self-regulation	Using professional organization as major referent	Belief in public service
Autonomy	1.00	0.268**	0.155*	− 0.129	− 0.032
Sense of calling to the field	0.268**	1.000	0.067	0.170*	0.112
Belief in self-regulation	0.155*	0.067	1.000	− 0.062	− 0.141
Using professional organization as a major referent	− 0.129	0.170*	− 0.062	1.000	− 0.070
Belief in public service	− 0.032	0.112	− 0.141	− 0.070	1.000

^**^Correlation is significant at the 0.05 level

^*^Correlation is significant at the 0.1 level

The construct ‘autonomy’ had a small correlation with ‘belief in self-regulation’ (0.155*) and medium correlation with ‘sense of calling to the field’ (0.268**). ‘sense of calling to the field’ also had a small correlation with ‘using professional organization as major referent’ (0.170*). ‘belief in public service’ had no significant correlation with any other constructs.

### Reliability and validity

The Cronbach’s alpha for the HPS constructs ranged between 0.52 and 0.72 (see Table 1), which was acceptable. Cronbach’s alpha for cognitive tests is 0.70, but when dealing with cognitive constructs such as professionalism, values below 0.70 would be foreseeable, owing to the diversity of the constructs being measured [17].

## Discussion

This study described the practically significant differences between the means of the HPS constructs and certain demographic variables. Demographics, among other factors, are important predecessor constructs of nursing professionalism [2]. The variables gender (item 1), undergraduate qualification (item 4), marital status (item 5) and employment sector (item 7) had no significant/practical association with any of the five HPS constructs. However, in international studies, gender [11, 16] and undergraduate qualifications, specifically higher educational degrees [10, 15], had an association with professionalism. Certain authors found that nurses pursuing bachelor’s degrees and higher were more attentive to professional values than nurses with lower educational levels [12, 13]. It is also noteworthy that nursing students’ professional values can be significantly transformed depending on the nursing curriculum they follow [18].

Studies among Korean and Iranian nurses respectively indicated that there was a relationship between professionalism and both age (item 2) and years’ of nursing experience (item 8), which was consistent with this study [6, 10]. In this study, qualified nurses aged 50 years and older and those with the most years’ of nursing experience had a higher professionalism score than those with fewer years of nursing experience. This finding is related to the outcome of the age of becoming a professional nurse (item 3), indicating that persons who had become professional  nurses after age 30 had higher scores of professionalism than those who qualified at a younger age. This could be due to maturity or years of nursing experience obtained in practice. The author could not specifically find a relationship between professionalism and the number of children nurses had (item 6), but a study in Ghana exploring work and family demands as predictors of work–family conflict found that nurses are at risk, which can result in weakened quality patient care [19]. This study, however, found that nurses with two and more children had a higher professionalism relating to ‘belief in public service’ than those with one child, which is a unique finding of this study because more children usually result in greater family demands. A study based on nurses from two international countries (United States and Korea) found different levels of professionalism [10]. This study links to the findings of the international study that international experience (item 9) played a role in nurses’ professionalism. Nurses with no international experience had more ‘autonomy’, whereas those with international experience had a higher ‘using professional organization as major referent’ score. A study conducted on Iranian nurses indicated that professionalism is affected by employment status (item 10) [6]. This was also true in this study as nurses employed full time scored lower in ‘autonomy’ and ‘belief in self-regulation’ than temporarily employed nurses. In addition, the variable of a nurse being satisfied with a nursing as a career (item 11) is related to professionalism [16], which improves the quality of patient care [6, 10]. Professionalism in nursing is also affected by the work environment, as nurses who have more autonomy in the workplace had higher levels of professionalism and job satisfaction than those working in hospitals, where job descriptions could be less clear [20].

## Limitations

- The population consisted of a small number of South African professional nurses, and thus the results cannot be generalised to the larger South African professional nurse population or to other countries.
- Using SMSs as a data collection method was not favourable due to low response rates.

## Data Availability

The author can confirm that all relevant data are included in the article.

## Abbreviations

HPS: Hall’s Professionalism Scale
SMS: Short message system
M: Mean
SD: Standard deviation
